# Individual Variability in *Bothrops atrox* Snakes Collected from Different Habitats in the Brazilian Amazon: New Findings on Venom Composition and Functionality

**DOI:** 10.3390/toxins13110814

**Published:** 2021-11-18

**Authors:** Leijane F. Sousa, Matthew L. Holding, Tiago H. M. Del-Rei, Marisa M. T. Rocha, Rosa H. V. Mourão, Hipócrates M. Chalkidis, Benedito Prezoto, H. Lisle Gibbs, Ana M. Moura-da-Silva

**Affiliations:** 1 Laboratório de Imunopatologia, Instituto Butantan, Av. Vital Brazil, 1500, São Paulo 05503-900, SP, Brazil; leijianes@gmail.com (L.F.S.); tiago.moretto@esib.butantan.gov.br (T.H.M.D.-R.); 2 Life Sciences Institute, University of Michigan, Ann Arbor, MI 48104, USA; matthewholding28@gmail.com; 3 Laboratório de Herpetologia, Instituto Butantan, Av. Vital Brazil, 1500, São Paulo 05503-900, SP, Brazil; marisa.rocha@butantan.gov.br; 4 Laboratório de Bioprospecção e Biologia Experimental, Universidade Federal do Oeste do Pará—UFOPA, Rua Vera Paz, s/n, Santarém 68035-110, PA, Brazil; mouraorhv@yahoo.com; 5 Laboratório de Pesquisas Zoológicas, Unama Centro Universitário da Amazônia, Santarém 68010-200, PA, Brazil; chalkidis@hotmail.com; 6 Laboratório de Farmacologia, Instituto Butantan, Av. Vital Brazil, 1500, São Paulo 05503-900, SP, Brazil; benedito.prezoto@butantan.gov.br; 7 Department of Evolution, Ecology, and Organismal Biology, Ohio State University, Columbus, OH 43210-1242, USA; gibbs.128@osu.edu

**Keywords:** *Bothrops atrox*, individual variability, venom heterogeneity

## Abstract

Differences in snake venom composition occur across all taxonomic levels and it has been argued that this variation represents an adaptation that has evolved to facilitate the capture and digestion of prey and evasion of predators. *Bothrops atrox* is a terrestrial pitviper that is distributed across the Amazon region, where it occupies different habitats. Using statistical analyses and functional assays that incorporate individual variation, we analyzed the individual venom variability in *B. atrox* snakes from four different habitats (forest, pasture, degraded area, and floodplain) in and around the Amazon River in Brazil. We observed venom differentiation between spatially distinct *B. atrox* individuals from the different habitats, with venom variation due to both common (high abundance) and rare (low abundance) proteins. Moreover, differences in the composition of the venoms resulted in individual variability in functionality and heterogeneity in the lethality to mammals and birds, particularly among the floodplain snakes. Taken together, the data obtained from individual venoms of *B. atrox* snakes, captured in different habitats from the Brazilian Amazon, support the hypothesis that the differential distribution of protein isoforms results in functional distinctiveness and the ability of snakes with different venoms to have variable toxic effects on different prey.

## 1. Introduction

Snake venoms are complex mixtures of toxic components belonging to multipleprotein families [1], each of which expresses several isoforms that are present in the venoms in different proportions [2]. The concentration of each isoform present in the venoms is highly variable and modulates venom function [3] and, as a consequence, the complexity of the venoms is associated with differences in their toxicity to a wide range of prey [4]. In addition, differences in venom composition are argued to be an adaptation that has evolved to facilitate the capture and digestion of prey and evasion of predators and is observed across all taxonomic levels, particularly in species with wide distributions [5,6].

*Bothrops atrox* (common lancehead) is a terrestrial pitviper widely distributed across the Amazon, from tropical lowlands to the rainforest of northern South America east of the Andes [7], where it occupies different types of habitats. Variability is widely reported in the composition of *B. atrox* venom and has been associated with ontogeny [8,9,10], geographical distribution [11,12,13,14], and environmental characteristics [15]. This species is responsible for most of the human envenomations in the Amazon region [16]. In envenomed patients, the intraspecific variability in *B. atrox* venom composition may hamper the patients’ prognosis as venom isoforms are involved in distinct clinical symptoms [17]. In some cases, functionally relevant isoforms present in high levels in venoms of a particular group of snakes may show lower reactivity with antivenoms and reduce the effectiveness of the treatment of some victims of snakebite [15,18].

Genetic differences among populations may correspond to the expression of different venom isoforms with distinct or similar functions [2,14]. However, venom variability also occurs among specimens from the same geographical areas [2] and may occur during the life span of individuals as ontogenetic variation related to an increase in body size, allowing capture and digestion of larger prey [19,20].

Large rivers in the Amazon basin contain ecologically diverse habitats and these have been associated with diversification in specific groups of vertebrates, such as birds [21]. In the west of Pará State, Brazil, the Amazon River is wide and has distinct habitats, such as upland forests on either bank and floodplain habitats. These represent distinct habitats that could generate diversification in venom phenotypes. For the last few years, our studies have been focusing on the variability in venom composition in this particular region of the Brazilian Amazon. Snakes have been collected at forest, pasture, and floodplain areas on both banks of the Amazon river. Using genome-scale RADseq data, we showed an interesting pattern of gene dispersal, suggesting a role for the Amazon River as a driver of in situ divergence both by impeding (but not preventing) gene flow and through parapatric differentiation along an ecological gradient [22]. The transcriptomes of the venom glands from the snakes collected in the northern or southern banks of the river denoted the same pattern of transcripts regarding the major toxin groups, but with the expression of different alleles or paralogs in snakes from the northern or the southern banks [2]. Using the comprehensive transcriptomic annotations described above, we compared the phenotype of pooled venoms from *B. atrox* snakes collected forest, pasture, or floodplain habitats by comparing the major toxic activities with data from free-label proteomics of the whole pools of venoms and the identification of relevant isoforms separated from these pools by RP-HPLC chromatographies, also by the proteomics of each fraction [15]. We observed two predominant phenotypes: pooled venoms from the forest, pasture, and degraded areas were more hemorrhagic, while the venom pool from snakes collected at the floodplain was more procoagulant. However, these analyses used pooled venom samples and so the level and significance of venom variation at the level of the individual snake are not understood. This information could help clarify the relative importance of habitat compared to individual variation as modulators of the venom variability observed in our previous report [15].

Here, using individual venom samples extracted from the snakes collected at the same habitats, and the proteomics information obtained previously, we investigated the hypothesis that the heterogeneity in venom composition could be higher in more unsettled environments. We found that the variability in the composition and functional activities is higher within individual venoms from snakes collected at the floodplain habitat, an extremely dynamic environment subjected to drastic seasonal changes, thus supporting our initial assumption. Moreover, our findings provide a deeper view of the main toxins and biological activities related to the individual venom variability within these *B. atrox* groups and suggest that the functional diversity of the venoms appears to be relevant to the ability of these snakes to persist in highly variable unsettled environments, such as the floodplain habitat.

## 2. Results

### 2.1. Individual Variation of Venom Composition Is Associated with Habitats

**Figure 1:**
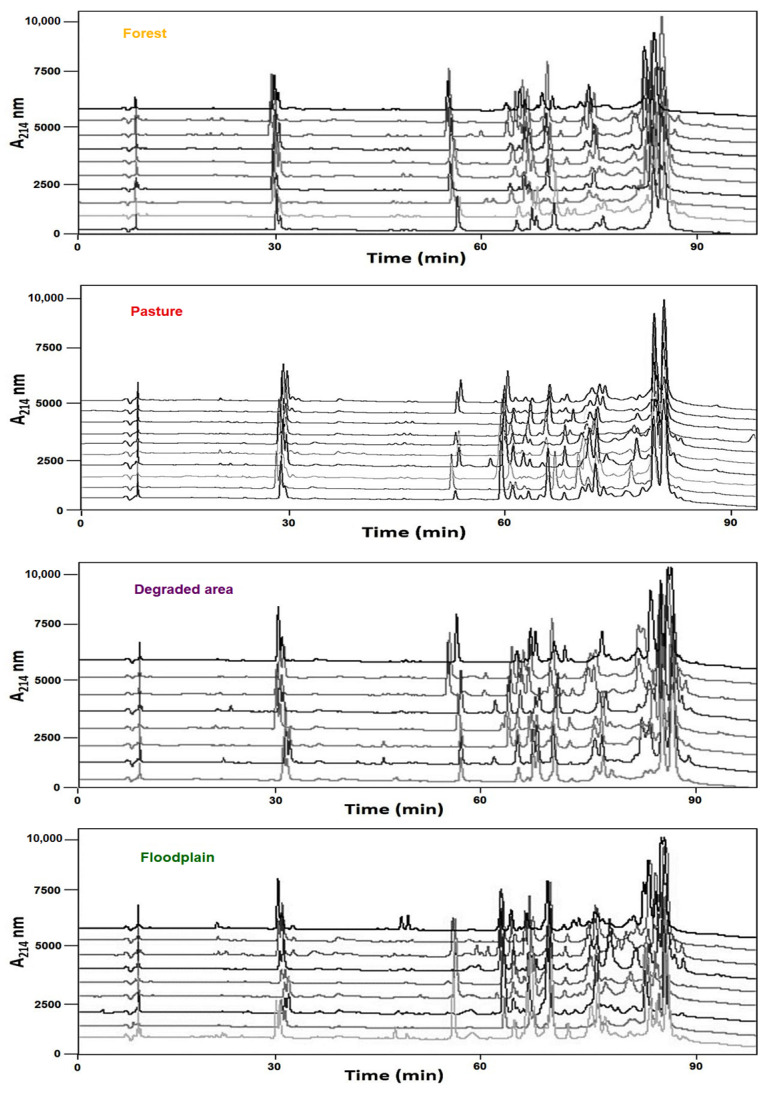
Chromatographic profiles of *B. atrox* venoms from different habitats west of Pará State, Brazilian Amazon. Individual venom samples (5 mg) of *B. atrox* snakes collected at the forest, pasture, degraded area, or floodplain were applied to a Vydac C-18 column. Mobile phases used were 0.1% TFA in water (solution A) or 0.1% TFA in acetonitrile (solution B). Proteins were gradient-eluted at 2 mL/min (5% B for 5 min, 5–15% B over 10 min, 15–45% B over 60 min, 45–70% B over 10 min, 70–100% over 5 min, and 100% B over 10 min). Separation was monitored at 214 nm.

Chromatographic analyses by RP-HPLC were our first approach to investigate the individual variability of *B. atrox* venoms among the specimens collected in each habitat: forest, pasture, degraded area, and floodplain. Overall, similar profiles can be seen in all chromatograms, although the relative heights or areas of peaks varied greatly between venoms even among snakes collected at the same habitat (Figure 1 and Supplementary Figure S1, for individual chromatograms). In all venom chromatographies, the highest peaks were eluted after 80 min, while peaks eluted between 50 and 70 min showed the most variable area percentages, being higher in floodplain venoms and lower in pasture venoms. According to our previous study [15], Snake Venom Metalloproteinases (SVMPs) are the main components eluted after 80 min, while Phospholipases A_2_ (PLA_2_s), C-type Lectin-like proteins (CTLs), and Snake Venom Serine proteinases (SVSPs) are the prevalent protein families in the fractions eluted in the intermediate steps (50–70 min).

### 2.2. Population-Level Differentiation between Spatially Disparate B. atrox Groups

**Figure 2:**
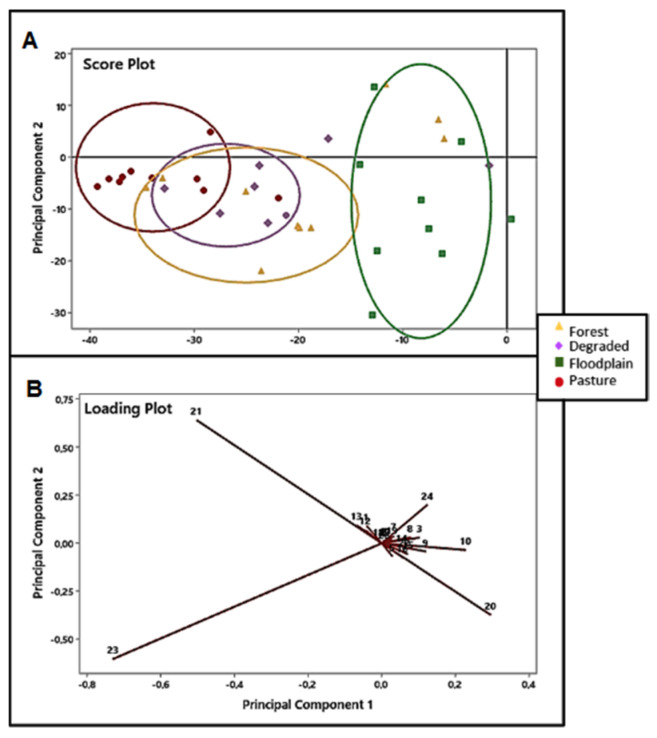
Principal component analysis based on peak areas of *B. atrox* individual venoms according to their chromatographic profiles by RP-HPLC, using a C-18 column. Score (**A**) and loading (**B**) plots in 2D graphs of the principal components axes (PC1 = 36% and PC2 = 22.5%) of venoms from *B. atrox* snakes captured in the forest (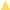), pasture (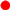), floodplain (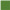), and degraded (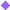) habitats.

First, we identified the population-level differentiation among venoms of snakes collected at the different habitats, based on the individual venom composition of each group of snakes. The PERMANOVA analysis (Table 1) showed that the environment explained 22.5% of the total variation in venom peak abundances in the sampled snakes (df = 3.33, F = 3.2, *p* < 0.0001). Further exploration of these differences with compositionally robust PCA (Figure 2, score plots) showed that PC1 largely differentiates floodplain from pasture venoms. Forest and degraded venoms occupying an intermediate space on the PC1 axis. Components with the most prominent loadings are fraction 10 and the fractions eluted after 85 min. The highest negative values of PC1, responsible for clustering the pasture venoms, were fraction 23 (PC1 = −0.7308, PC2 = −0.6035) and fraction 21 (PC1 = −0.5028, PC2 = −0.6410). These fractions were characterized in our previous study [15] as PIII-class and PI-class SVMP isoforms, respectively. The highest positive values of PC1, responsible for clustering the floodplain venoms, were fraction 20 (PC1 = 0.2958, PC2 = −0.3732), fraction 24 (PC1 = 0.1241, PC2 = 0.2006), and fraction 10 (PC1 = 0.2284, PC2 = −0.0353), which in our previous study have been characterized as different isoforms of the PIII-class and PI-class SVMPs, with minor proportions of CTL isoforms, and an SVSP, respectively (Figure 2, loading plots).

In addition, posthoc comparisons of individual population pairs using PERMANOVA showed that the spatially proximate degraded and forest habitats were the only locations between which venom composition did not significantly differ. All other population pairs differed significantly, with the greatest degree of differentiation between the spatially disparate floodplain and pasture populations.

**Figure 3:**
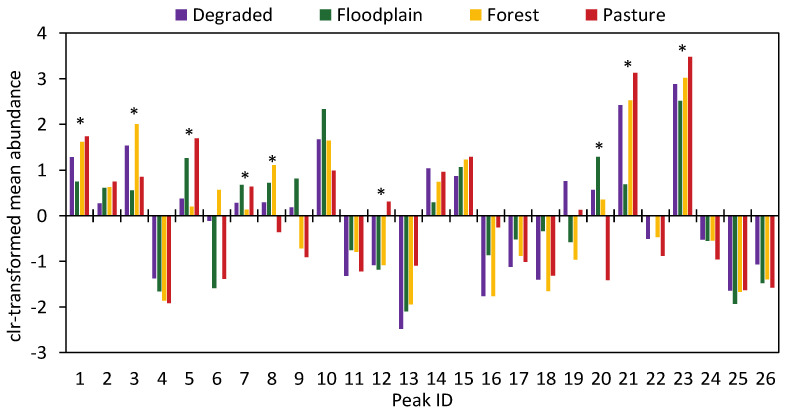
Variation in the chromatographic fractions in *B. atrox* snakes from different habitats. The centered log-ratio mean abundance for each reversed-phase high-performance liquid chromatography peak was plotted. Negative numbers correspond to low-abundance peaks, whereas positive numbers correspond to high abundance peaks. (*) Asterisks indicate peaks that show significant population-level variation. X-axis labels correspond to RP-HPLC peak numbers (1 to 26).

Next, peak-by-peak comparisons showed that several chromatographic peaks contributed to population-level differentiation among venoms. After FDR-correction, nine peaks showed significant variation: Peaks 1, 3, 5, 7, 8, 12, 20, 21, and 23 (Figure 3).

In terms of comparisons between habitats, venoms from the forest or recently degraded area were similar in relative peak abundances. Pasture venom was distinct with a higher abundance of Peaks 5 and 7, which correspond to the acidic PLA_2_s, Peak 12, with CTL as the major toxin, and Peaks 21 and 23, which include the SVMPs. Significantly lower abundances were observed in pasture venoms for Peaks 8 and 20, which have as major toxins PLA_2_s and PI-SVMPs, respectively. Peak 20 was practically absent in pasture venoms (Figure 1). Floodplain venoms were distinct in peaks related to SVMPs and PLA_2_s: Peaks 1, 21, and 23, which contain SVMPs and disintegrins, were present at a lower abundance, while Peak 20, which contains mostly PI-SVMPs, is at a comparatively higher abundance. In peaks containing PLA_2_s, Peak 3, which contains K-49 basic PLA_2_s, is less abundant, while Peak 7, which contains acidic PLA_2_s, and Peak 8, rich in D-49 basic PLA_2_s, were proportionally higher in the venoms from the floodplain.

### 2.3. Venom Differentiation Is Not Limited to Rare (Low Abundance) Proteins

**Figure 4:**
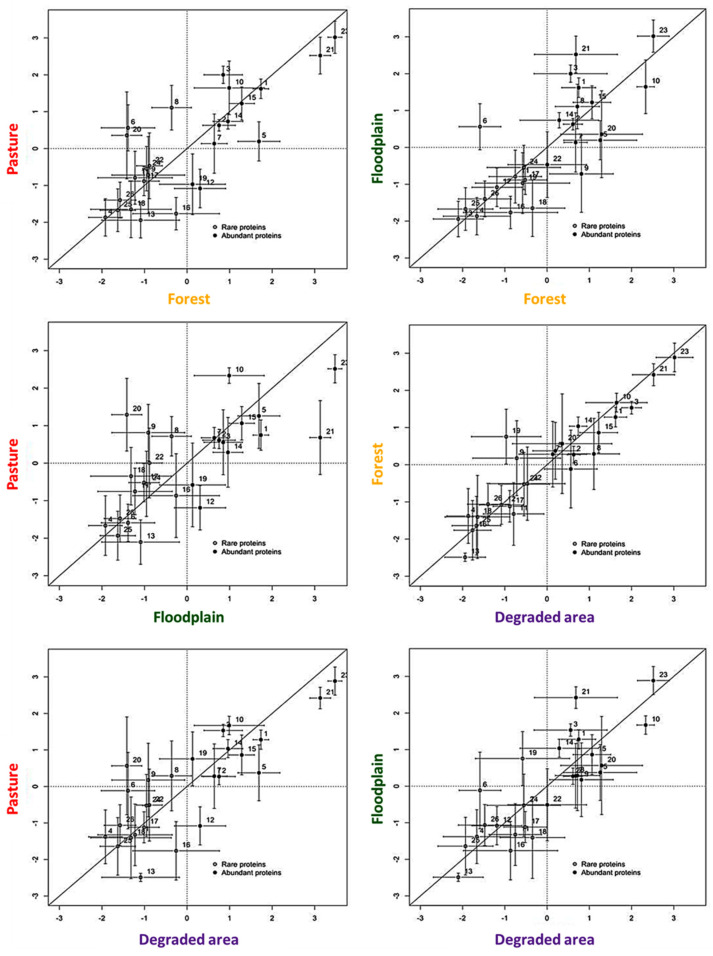
Comparisons of the degree of differentiation in low vs. high abundance proteins based on chromatographic peak areas from *B. atrox* individual venoms. The clr mean was plotted for each RP-HPLC peak across the different habitat (x-axis) and (y-axis) populations for *B. atrox* snakes. Bars indicate the SE, a solid line indicates a perfect agreement, dashed lines indicate the origin (i.e., the geometric mean), and proteins less than these values were considered low-expression proteins.

We also investigated whether the venom variability in *B. atrox* groups would be associated with the degree to which the venom proteins were expressed in the different habitats. RP-HPLC peaks for each habitat were classified into two sets: low or high abundance, based on the clr-transformed mean of each peak and a nonparametric MANOVA analysis, as previously described [23], where rare proteins = the mean of an individual peak < the geometric mean, and abundant proteins = mean of an individual peak > the geometric mean (Figure 4). Proteins showing deviations off the middle line are those in which the expression levels are different between the compared populations whereas those that are on the line are similar.

In our PERMANOVA analyses, only in the spatially proximate degraded and forest habitats the venom composition did not significantly differ (Table 1). Accordingly, minor differences in both rare and abundant proteins were observed between these populations, which showed only slight differences in the rare proteins. However, in the populations from the other habitats, the venom differentiation was not constrained to rare proteins. On the contrary, in some cases, these proteins showed more similarity across populations from different habitats (for example floodplain vs. forest; pasture vs. forest) than proteins classified as abundant. In fact, except for peak 23 (rich in PIII-class SVMPs), abundant proteins showed differences across all habitats. Interestingly, the abundant proteins would be closely related to the functional activity of the venom toward their prey. As shown before [15], the conserved fraction 23 contains Batroxrhagin, a multifunctional PIII-class SVMP extremely conserved in different samples of *B. atrox* venom [2]. However, in Figure 4, we show that other abundant toxins are differentially expressed between the snakes from different habitats: fraction 21, which predominantly contains a PI-class SVMP, had lower expression in floodplain venoms; fraction 10, which contains mostly SVSPs, had higher expression in floodplain venoms; and fraction 20, which predominantly contains a PI-class SVMP and CTLs, was relatively over-expressed in venoms from pasture compared to the other areas.

### 2.4. Specific-Level Differentiation among Venoms of Snakes Collected in the Same Environment

Once the variability among the groups was defined, we quantified intrapopulation variability in venom composition, among specimens collected in close proximity in the same areas. As shown in Figure 1, HPLC chromatograms show variable profiles within venoms from snakes collected in the same environment (for more details see Supplementary Figure S1). Consistent with this pattern, we found great heterogeneity in the percentage area of each peak in the groups (Table 2).

In every habitat, individual venoms showed important differences in both their percent area and the presence/absence of some peaks. Venoms from pasture and floodplain snakes were again the most distinct among the individuals from the same group (Table 2; Supplementary Figure S1), with venoms from the floodplain being the most heterogeneous. From Table 2 we emphasize three regions: In the PLA_2_-eluting fractions (Peaks 3 and 5), Peak 3 was absent in several chromatograms, but its absence is offset by the homogeneous increase of Peak 5 in pasture specimens. Venoms from floodplain specimens showed the most heterogeneous distribution of both PLA_2_ fractions. CTL fractions (Peaks 15–17) are expressed at low but homogeneous levels in pasture venoms with increased variation in venoms from the forest and degraded area, reaching the highest heterogeneity in venoms from the floodplain. A notable observation is the high level of variation in the expression of the peaks containing SVMPs (peaks from 21 to 23) in floodplain specimens. These are the most abundant and homogeneous fractions in venoms from the other areas, but in venoms from the floodplain, fraction 21 is very low or even absent in venoms of five specimens while fraction 22, almost not present in venoms from other areas, is detectable in high levels in venoms from four specimens from the floodplain, indicating a high variety of SVMP isoforms venoms from snakes in this environment. It is important to note that the higher variability observed in venoms from floodplain snakes is not due to the two distant floodplain spots of snake collection. The differences appointed here can be observed within the snakes collected at Santarém (ATXV 5, 7, 8, 9, and 16) or Oriximiná (ATXV 10, 11, 12, and 13), which are approximately 300 km apart.

### 2.5. Differences in the Composition of Individual Venoms Resulting in Functional Variability

The most variable components among the venoms included SVMPs, SVSPs, and PLA_2_s. Our next step was to evaluate some of the main biological activities related to these protein families. With the goal of reducing the number of experimental animals for toxicity tests for ethical reasons and due to the limited amounts of some individual venom samples, venoms from only 16 specimens were evaluated per functional test, comprising four from each habitat.

**Figure 5:**
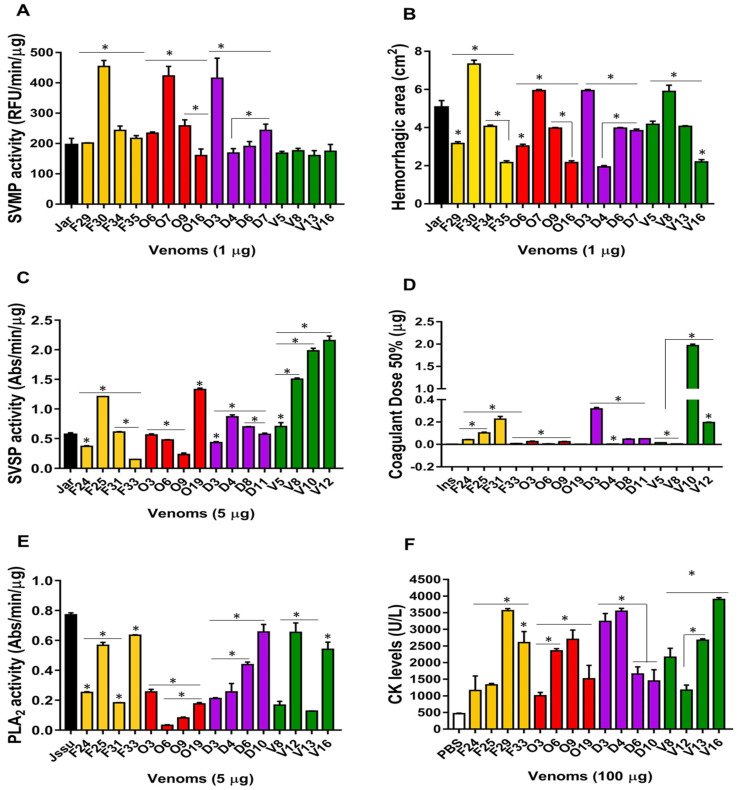
Functional assays: (**A**) SVMP catalytic activity: evaluated by hydrolysis of FRET substrate (Abz-AGLA-EDDnp), expressed as RFU/min/μg of venom. (**B**) Hemorrhagic activity: evaluated by the size of the lesions observed 3 h after venom injection (10 μg) into the dorsal skin of mice, and expressed in cm^2^. (**C**) SVSPs catalytic activity: evaluated by hydrolysis of the chromogenic synthetic substrate (L-BAPNA), and expressed in Abs/min/μg of venom. (**D**) Pro-coagulant activity: evaluated the clotting times measured by thromboelastography of recalcified plasma from chickens, and the results were expressed in terms of Coagulation Dose 50% (CD_50_). (**E**) PLA_2_s activity: evaluated by hydrolysis of the chromogenic substrate (NOBA), and the results were expressed in Abs/min/μg of venom. (**F**) Myotoxic activity: evaluated by the creatine kinase activity in mice serum 3 h after venom injection, and the results expressed in U/L. The data shown represent the mean + SD of three independent experiments. Controls: PBS and venom pools from *Bothrops jararaca*—Jar; *Bothrops jararacussu*—Jssu; *Bothrops insularis*—Ins. (*) Asterisks indicate significant variations among venoms from a same habitat.

As shown in Figure 5, individual variation was observed in the catalytic activities of the major enzymatic components (SVMPs, SVSPs, and PLA_2_s) among the venom samples from each habitat (Figure 5A,C,E), except in the venoms from the floodplain, in which the SVMP catalytic activity was similar and low. There were significant differences among the venoms collected at the same habitat in hemorrhagic (Figure 5B) and myotoxic (Figure 5F) activities. The four venoms from the floodplain snakes (V5, V8, V13, and V16) induced hemorrhagic spots comparable to those induced by snake venoms from the other habitats, with an emphasis on the V8 snake, whose venom had the highest hemorrhagic activity in the floodplain group. For myotoxic activity, the greatest variation was found among the venoms from floodplain snakes: this group was the only one in which all tested venoms showed statistically significant differences in activity (Figure 5F).

The greatest within-group difference was found in procoagulant activity among the floodplain snakes, in which the DC_50_ values varied from 0.0035 to 1.965 μg for V8 and V10 snakes, respectively (Figure 5D). No other group showed such a large variation in DC_50_ values. However, the wide range of activity in this group was mostly due to the very high DC_50_ value of only one specimen (V10). On the other hand, pasture venoms were similar in procoagulant activity with only a small amount of variation observed in the venoms from the forest and degraded areas.

### 2.6. Individual Heterogeneity in Venom-Induced Lethality

**Figure 6:**
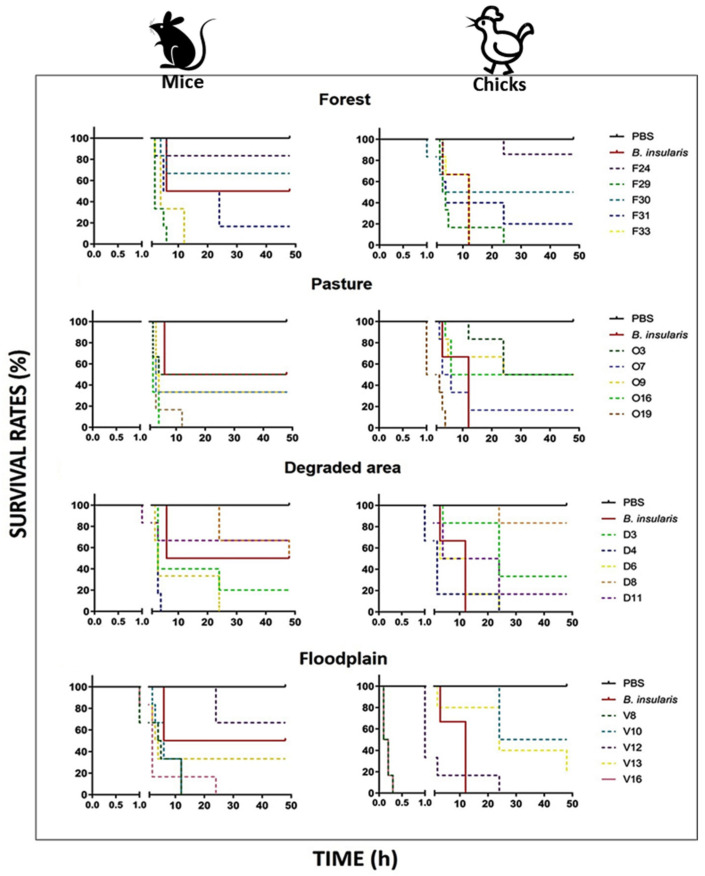
Time of death induced by individual *B. atrox* venoms in avian and murine models. Single doses of selected venom (200 μg) were injected i.p. into groups of six animals (mice or chicks). Next, the deaths were followed up every hour until 6 h after, then at 12, 24, and 48 h after venom injection. The data are representative of two independent experiments.

Finally, to link functional variation to toxicity to specific prey, we decided to investigate the ability of the venoms from each habitat to kill rodents (mice) or birds (chicks). We also quantify individual variation in venom-induced lethality within the same habitat. Venoms from five specimens from each habitat were injected into groups of six mice or six chicks, and the time of death of the animals was monitored over 48 h (Figure 6).

We observed a great deal of variation in the lethal activity of venoms from individual venoms from all groups analyzed. In venoms from the forest, degraded area, and pasture areas, mortality of experimental animals was observed from 1 to 2 h after venom injection, while in a number of cases, several animals survived through 48 h of observation. Similar results were observed for both the mice and birds, which matches the generalist diet preferences of ***B. atrox***. There were some quite interesting patterns of toxicity in floodplain venoms. These venoms had similar activity to venoms from the forest, pasture, and degraded area when injected in mice. However, some venoms from the floodplain habitat (V8 and V16) induced deaths only a few minutes after its inoculation in chicks, a pattern that was not observed in mice. In addition, one venom (V12) was noticeably more lethal to chicks, killing half the animals in the first two hours and all animals of the group within 24 h, whereas in mice, the same venom induced the death of only two animals, 12 and 24 h after inoculation. However, not all floodplain venoms were highly lethal to birds. The V10 snake venom was much more lethal to rodents than to birds, killing all mice within 24 h, and only two chicks until the end of the experiment. The venom of snake V13 killed the same number of chicks and mice (only 2), although at different time intervals. Similar to the variations observed in the venom composition, the differential lethality to chicks observed with floodplain venoms was not due to the two distant floodplain spots of snake collection. Venoms that preferentially killed chicks were from snakes collected at Santarém (ATXV 8 and 16) or Oriximiná (ATXV 12 and 13).

## 3. Discussion

In this study, we analyzed the influence of different environments on the individual variability of venom samples obtained from *B. atrox* snakes captured in four different habitats of the Brazilian Amazon: forest, pasture, degraded area, and floodplain. Our analyses revealed clear differences in both venom composition and its biological activities among snakes from different habitats. However, major differences were also observed among venom samples collected from snakes in the floodplain habitat, a dynamic environment, subject to periods of annual drought and floods.

In a previous report involving *B. atrox* snakes, the variability in venom composition was attributed mainly to low-abundance proteins that would be a genetic reservoir for quick adaptive changes [2], while the most abundant isoforms from each toxin family were considered as “core toxins”, conserved in venoms of most specimens and responsible for the major activities of the venom [2]. A similar observation was reported by Margres and collaborators [23], showing that, in *Crotalus adamanteus*, *Sistrurus miliarius*, and *Agkistrodon piscivorus* snakes, the differences in the expression levels were present mostly in the low-expression proteins. However, in the present study, both rare (low expression) and abundant (high expression) proteins show contribute to venom variation within and between snakes from different habitats.

For example, our data also showed that Peak 23 was composed mostly of Batroxrhagin, a P-III class SVMP from *B. atrox* venom isolated by our group [24], was widely conserved in all the 37 venoms analyzed, within and across all groups; this confirms a previous observation [2] that Batroxrhagin would act as a “core toxin” in *B. atrox* venoms, acting on important physiological targets of diverse prey types. In contrast, other abundant venom proteins showed less conservation and could represent more specific or even “adaptive” variation in these proteins. Clear examples of abundant fractions differentially expressed between the groups are fraction 21, which contains predominantly a PI-class SVMP and had lower expression in floodplain venoms; fraction 10, which contains mostly SVSPs and had higher expression in floodplain venoms; and fraction 20, which contains predominantly a PI-class SVMP and CTLs and is higher expressed in venoms from pasture compared to the other areas. Other fractions rich in SVSPs and CLTs were also were variable. These proteins participate to the hemorrhagic and coagulant activity of *B. atrox* venom and the observed differences in expression of the isoforms may alter procoagulant activity and to promote differential lethality in rodents and birds, suggesting an important role of the environment in selecting for venom variation.

Individual variation in the abundance of SVMPs, SVSPs, and PLA_2_s could have arisen as a result of environmental differences between habitats. In our previous study [15], we compared the functional profiles of pools of venoms from *B. atrox* snakes from the same habitats and observed a less hemorrhagic and more procoagulant phenotype in the pool of venoms from floodplain snakes. The pool of venoms from the floodplain snakes was also significantly faster to induce clotting in several types of plasmas, including avian plasma [25]. Here, we used similar experimental approaches to investigate the individual variability within each group of snakes, introducing modifications in the tests of DC_50_, which were performed in the presence of calcium, to ensure the detection of coagulotoxins, dependent or not on this cofactor. In addition, our tests were performed with avian plasma as previously reported [26,27], which allowed us to construct better dose–response curves to assess the procoagulant effects of the individual venoms.

Venoms from *B. atrox* specimens from the floodplain showed low SVMP activity and higher SVSP catalytic activities. However, the hemorrhagic and procoagulant phenotypes observed in the pool of venoms were not consistent across all individual venoms collected from this environment. Some individual venoms such as from V5 and V8 snakes, showed both a potent procoagulant action on avian plasma and high hemorrhagic activities in mice. Moreover, the higher DC_50_ value observed, which corresponds to the less procoagulant venom, was precisely from a floodplain snake venom (V10), demonstrating that not all snakes from the floodplain have venoms that induce potent clotting of avian plasma, but the range of clotting activity was the highest in this environment.

Bernardoni and collaborators [3] analyzed *Bothrops neuwiedi* venom and found SVMPs isoforms that are functionally different and capable of affecting different targets in the hemostatic systems of birds, rodents, and humans. They suggested that some SVMPs are less selective, guaranteeing the action of the venom on different targets, while other isoforms are more selective, modulating the action of the venom for specific prey. More recently, we also investigated the effects of these SVMP isoforms on amphibian plasma (*Rinella marina*), demonstrating the coagulotoxic effects of these toxins on different types of animal plasmas [18]. In the present study, some fractions rich in SVMPs had a very variable distribution in the *B. atrox* venoms of all habitats and may be involved in the variability observed in the coagulotoxic activity observed in these individual venoms. In contrast, the major SVMP eluted in Peak 23 was very conserved in the venoms of all 37 specimens analyzed, confirming the previous assumption [2] that Batroxhagin is the core SVMP of *B. atrox* venom. On the other hand, the strong hemorrhagic action observed in some floodplain venoms (V5, V8, and V13), is compatible with a prominent increase of the fractions represent by Peaks 21 and 22 that shows height/area comparable to the ones observed in venoms from the other habitats. These peaks contain Atroxlisin-Ia, a PI-class SVMP that induces hemorrhage comparable to the P-III class enzymes [28].

During envenomation, various components present in snake venoms can act synergistically to cause the prey’s organism to collapse, which usually results in rapid death. The speed to kill/immobilize prey is crucial for food acquisition of terrestrial viperids during hunting [29,30]. Previous works also report that lethality profiles of snake venoms can be variable in different animal models, such as mammals, birds, reptiles, and amphibians [5,31]. For this reason, we included the functional characterization of the individual venoms in two different animal models, birds (chicks) and mammals (mice). We found substantial differences in venom activities in both, the number of test animals dying, and the time of deaths in the two models. Forest venoms showed similar lethality profiles in rodents and birds across individual venom samples, while venoms from pasture and degraded areas were slightly more lethal to rodents. Of particular note, venoms from floodplain snakes were highly variable according to their ability to prey on mammals or birds. For example, the venoms of the snakes V8 and V16 were more toxic to chicks, inducing deaths within few minutes in birds, even more quickly than the venom of *B. insularis*, a snake with a diet specialized in birds [32]. The venom of the snake V10 however was more lethal to rodents. Interestingly, the venoms with the highest procoagulant activity are more lethal to birds while V10 venom, the weakest procoagulant, killed predominantly mice. The limitations imposed by the small available quantities of venom prevented using the same individual venoms in all the functional tests performed or to carry out dose–response analyses, hindering a more direct assessment of the relationship between lethality and the other toxic activities evaluated.

Nevertheless, our findings strongly suggest that the procoagulant activity of the floodplain venoms contributes significantly to their lethal effects in rodents and birds. The most striking and differential characteristics of the floodplain venoms are its potent effects on coagulation in different types of plasma. The venom of the snake V10 (from floodplain habitat) was more efficient in killing rodents than birds, and this same venom had the highest EC_50_ value on avian plasma. A possible explanation for the remarkable action of some floodplain venoms on hemostasis and velocity to induce death in birds could be directly linked to the floodplain environment, where these snakes were captured. Floodplains are periodically flooded by the lateral overflow of waters rich in sediments from the Amazon River. The floodplain regions alternate cyclical periods of drought and flood, remaining flooded for a few months during the year, this seasonal variation being driven by flood and precipitation seasons [33]. Balancing selection pressures could result in greater venom heterogeneity, as evidenced by the differential lethality to rodents or birds observed in some floodplain venoms, while the snake venoms from the other habitats presented a more homogeneous lethality pattern.

In line with our findings, Smiley-Walters et al. [31] showed that variation in the venom of *Sistrurus miliarius* at the population level has an adaptive role in terms of toxicity to prey. Testing adaptive hypotheses requires careful analysis of phenotypic characters whose variation has clear functional consequences. Venoms directly affect the ability of an individual snake to immobilize and kill its prey [31,34,35,36,37]. For *B. atrox*, a snake with a generalist diet that includes arthropods, frogs, lizards, birds, and small mammals [38,39], the functional diversity of the venoms could represent a local adaptation of individual venoms in each population to different sets of prey. Within an ecological context, the nature of the interactions between species, including prey–predator relationships, may change among populations [40,41], especially for species with a wide geographical distribution. of the functional versatility and diversity of *B. atrox* venoms may explain its wide distribution throughout all Amazon regions [7], reflectinghow this species adapts to prey communities in different habitats.

## 4. Conclusions

Individual venom variation of *B. atrox* snakes, captured in different habitats within the Brazilian Amazon, support the idea that dynamic environments may select for more variable venoms. Such differential distribution of protein isoforms leads to differential function and toxic effects on different prey. The fact that the greater heterogeneity in terms of composition/toxicity of the venom was found in snakes from the floodplain habitat suggests that balancing selection for expression of different isoforms could be enacted by the drastic seasonal changes (drought/flood) present in this type of environment. Variable selective pressures in different habitats may likely exert localized impacts on the venom phenotype in a species with a wide geographic distribution, such as *B. atrox*.

## 5. Material and Methods

### 5.1. Snakes and Venoms

Thirty-seven male and female adult specimens of *B. atrox* snakes, with sizes ranging from 71.2 to 124.5 cm, were captured in four environments at Santarem and Oriximiná, in the western region of the state of Pará, Brazil (Supplementary Figure S2) under ICMBio/SISBio license 32098-1 and SISGEN number A78BD88: (1) Pasture: ten adult snakes were collected from a pasture area in the municipality of Oriximiná (ATXO 1, 2, 3, 5, 6, 7, 9, 15, 16, 19) on the north shore of the Amazon River. This site was historically Terra Firme forest characterized by large trees that were cleared for pasture ~20 years earlier. (2) Floodplain (ATXV): five adults were collected from a seasonally flooded island in the main course of the Amazon river near Santarem (at Urucurituba: ATXV 5, 7, 8, and 9; at Tapará: ATXV 16) or Oriximiná (ATXV 10, 11, 12, 13). These areas are typical of floodplain habitats subject to periodic flooding by the Amazon River and formed by the deposition of sediments that have led to the formation of many islands. The typical vegetation consists of grasses that grow on highly fertile alluvial soils. (3) Forest: ten snakes were captured at the Floresta Nacional do Tapajós, a protected area located in the municipality of Belterra (ATXF 24, 25, 26, 28, 29, 30, 31, 33, 34, and 35) next to the Tapajos River about 50 km south of the Amazon River. This site also represents the upland Terra Firme forest. (4) Degraded: eight snakes were collected at a recently degraded (ATXD 3, 4, 6, 7, 8, 9, and 10) area contiguous to the forest area at Floresta Nacional do Tapajós, which was cleared for pasture. After capture, the snakes were transferred to the Herpetarium of Laboratório de Pesquisas Zoológicas, Universidade da Amazônia (UNAMA), in Santarém, PA. Venom samples were collected using manual extraction techniques, freeze-dried, and kept at −20 °C until use. Animal care and procedures used in the handling of snakes were undertaken according to the guidelines and permits (CEUAIB 1244/14, Instituto Butantan, São Paulo, Brazil).

### 5.2. Chromatographic Analysis

The *B. atrox* venom samples were individually fractionated by reverse-phase high-performance liquid chromatography (RF-HPLC) in the Vydac C18 column as previously described [15]. The fractions had their toxin composition predicted according to peak shape and retention time comparing to a previous standard chromatography of *B. atrox* venom from snakes collected in the same areas from which components present in each fraction were identified and quantified by free-label mass spectrometry [15].

### 5.3. Functional Assays

#### 5.3.1. Enzymatic Assays on Synthetic Substrates

SVMP, SVSP, and PLA_2_ enzymatic activities of individual venoms from *B. atrox* were assayed using synthetic substrates according to the procedures previously standardized in our lab [42]. Briefly, the substrate used for SVMPs was Abz-AGLA-EDDnp substrate (Peptide International, Gardner, MA, USA), and the results are expressed as Relative Fluorescence Units-RFU/min/µg. The PLA_2_ activity was assayed using the substrate 4-nitro-3-[octanoyloxy] benzoic acid (Enzo Life Sciences, New York, NY, USA) and activity were determined according to the absorbance at 425 nm and expressed as Absorbance/min/μg of venom. For SVSP catalytic activity, the chromogenic synthetic substrate benzoyl-arginyl-p-nitroanilide (L-BAPNA) (Sigma-Aldrich^®^, St. Louis, MO, USA) was used and the hydrolysis of the substrate was expressed as the increase in Absorbance/min/μg of venom. The results represent the mean ± SD of three independent experiments, undertaken in triplicate.

#### 5.3.2. Procoagulant Activity

The procoagulant activity was evaluated in recalcified chicken (White leghorn) plasma, obtained under license CEUAIB n° 13,710-14, Instituto Butantan, by thromboelastometry using a four-channel ROTEM^®^ system (Pentapharm, Munich, Germany), as previously described [26]. The plasma was prepared as 3.2% citrated stock and stored at −80 °C until needed in 1 mL aliquots. For the experiments, an aliquot was defrosted by placing it into a 37 °C water bath for 10 min. Venom samples and controls were dissolved in PBS (60 μL) and added in the specific cups with 20 μL of CaCl_2_ (0.02 M) and 260 μL of citrated plasma (final volume = 340 μL), pipette-mixed, and the clotting time was immediately recorded. PBS and ellagic acid were used as negative and positive controls, respectively. Under such conditions, recalcified chicken plasma treated with PBS only is unclottable for at least 30 min (CT value = 1800 s). The results are shown as Coagulant Dose 50% (DC_50_), defined as the amount of venom (μg) that reduces the CT parameter to 900 s.

#### 5.3.3. Venom Activities Using Animal Models

Hemorrhagic and myotoxic activities were carried out as previously described [43] using male Swiss mice (18–20 g) under the approval of the Butantan Institute Ethics Committee on Animal Use (Protocol Number: 13710-14). Briefly, for hemorrhagic activity, samples containing 10 µg of each venom pool, diluted in 50 µL of PBS, were injected intradermally into the dorsal skin of mice. After 3 h, the animals were euthanized in a CO_2_ chamber, the dorsal skin was removed, and the hemorrhagic halos were measured. Groups of 5 animals were tested, and control group animals were injected with PBS only or *B. jararaca* venom (10 µg). Results represent the mean ± SE of the area of hemorrhagic spots (cm^2^) from mice tested in at least 2 independent experiments. The myotoxic activity was assayed using 50 µg of venom pools in 30 µL of PBS, injected intramuscularly into the gastrocnemius muscle in Swiss mice. After 3 h, the animals were bled via ophthalmic plexus and the sera were assayed for creatine-kinase activity with a commercial kit CK-UV (Bioclin), according to the manufacturer’s instructions. Groups of 5 animals were tested, and animals from the control groups were injected with PBS only or *B. jararacussu* venom (50 µg). Results represent the mean ± SE of the CK activity in mice sera (U/mL) from mice tested in at least 2 independent experiments.

The lethality induced by the venoms was tested in murine (Swiss mice 18–20 g body weight) and avian (three days old Bovan chicks 20–30 g body weight) models with protocols approved by the Animal Ethical Use Committees of the Instituto Butantan (CEUAIB n° 13710-14). Groups of 6 mice or chicks were injected intraperitoneally with a single dose (200 μg) of venom samples, diluted in 500 μL of PBS. Following injection, the times of death for each group were recorded hourly until the sixth hour, and then at 12, 24, and 48 h after inoculation of the samples. After 48 h, the surviving animals were euthanized, using a CO_2_ chamber for the mice and injection of sodium pentobarbital overdose for the chicks. The data obtained were graphed on a survival curve plot. The tests were undertaken in two independent experiments.

### 5.4. Statistical Analysis

We tested for significant compositional differentiation using a hierarchical multivariate approach that is robust to the compositional nature of the data [44]. First, we brought the relative abundance data out of the compositional simplex space using the isometric log-ratio (ilr) transformation, which also avoids the zero-sum constraint of the centered-log ratio (clr) transformations but at the cost of one dimension of the data. The ilr-transformed peak data were then subjected to permutational multivariate analysis of variance based on distances using the adonis function from the vegan R package [45], with all peak dimensions as a response and environment as a predictor. Significance and R^2^ were estimated using 10,000 permutations of the raw data. We then visualized the venom phenotype of each snake in multivariate space using robust principal components analysis (PCA) as implemented in the pcaCoDA function from the robCompositions R package [46].

After detection of a significant global association between environment and venom composition, we conducted pairwise posthoc PERMANOVA, as described above, between the venom compositions of all pairs of environments to determine which environments differed significantly. Lastly, we preserved the full dimensionality of the data but removed the data from the simplex using the clr-transformation, to determine which peaks specifically varied across environments. To do this, we use the lm function from R stats, with the clr-transformed abundance of an individual peak as the response and environment of origin as the predictor. We repeated this test for all 26 peaks and used the false-discovery rate (fdr) approach to correct the *p*-values for multiple tests.

For the functional analyses (in vitro and in vivo assays), firstly, the data were evaluated for a normal distribution (Shapiro–Wilk), and then differences among the means were evaluated by one-way ANOVA, followed by a Tukey post-test (for multiple comparisons). Data that did not meet the normality criteria were analyzed using a non-parametric test (Kruskal–Wallis). Results represent the mean and standard deviation or standard error, as appropriate, and the level of significance was set at *p* ≤ 0.05. Data were analyzed using the GraphPad Prism statistical program (version 7.00 for Windows, GraphPad Software, San Diego, CA, USA).

## Figures and Tables

**Table 1:** Posthoc pairwise PERMANOVA analyses were used to test for significant venom compositional differentiation among all pairs of populations.

	Forest	Pasture	Floodplain	Degraded Area
Forest		<0.0001	<0.0001	0.34
Pasture	19.4		<0.0001	<0.0001
Floodplain	16.0	23.2		0.01
Degraded area	6.4	17.4	12.2	

Values shown are *p*-values (above diagonal) and R2 values (below the diagonal) for each comparison.

**Table 2:** Comparison of the chromatographic peak areas (%) from *B. atrox* individual venoms according to the habitat.

Area	Snake	Peaks (%Area)																									
		1	2	3	4	5	6	7	8	9	10	11	12	13	14	15	16	17	18	19	20	21	22	23	24	25	26
**Forest**	**F 24**	6.15	1.72	7.11	0.00	0.00	0.00	0.20	4.81	1.68	5.38	0.36	0.00	0.78	1.44	6.30	0.00	0.92	0.00	0.24	0.00	23.64	0.00	32.79	0.00	0.00	0.00
	**F 25**	4.54	2.15	7.84	0.00	0.00	0.00	0.59	5.04	3.83	9.77	0.64	0.00	0.00	4.40	3.47	0.00	0.59	0.00	1.97	10.29	12.28	0.00	29.10	0.00	0.00	0.70
	**F 26**	4.68	2.19	8.12	1.02	0.00	0.00	9.06	1.24	6.49	3.98	1.20	0.00	0.00	4.53	7.61	0.31	0.75	0.00	0.30	9.38	19.40	0.00	5.57	7.04	0.45	1.17
	**F 28**	5.20	2.89	6.02	0.00	0.00	0.00	0.60	3.15	2.93	8.83	0.30	0.00	1.79	6.51	0.00	0.00	0.39	0.28	0.69	16.06	13.16	0.00	27.72	0.00	0.22	0.00
	**F 29**	5.65	2.11	12.00	0.00	0.00	0.00	0.53	5.62	9.84	8.93	1.04	0.00	0.00	2.38	6.73	0.68	0.43	0.22	2.25	0.13	12.96	10.32	8.23	3.88	0.92	0.00
	**F 30**	5.96	2.70	9.88	0.00	0.00	0.00	0.28	4.70	3.55	6.26	0.33	0.00	1.76	1.19	0.79	0.00	0.67	0.16	2.05	14.50	12.61	0.00	28.62	0.00	0.00	0.00
	**F 31**	6.63	1.81	6.69	0.00	0.00	0.00	0.54	3.78	0.94	9.28	0.63	0.00	1.29	4.85	0.00	0.00	0.72	1.56	0.72	0.00	23.17	0.00	35.78	0.00	0.00	0.00
	**F 33**	6.14	1.65	9.98	0.00	0.97	0.91	5.08	0.24	7.66	0.00	0.80	0.00	0.00	0.00	3.48	1.18	0.00	0.71	0.00	1.75	1.12	14.45	36.50	0.00	0.00	0.79
	**F 34**	3.65	2.18	4.78	0.00	0.00	0.00	0.68	5.03	6.06	12.76	1.45	2.07	2.91	0.26	0.00	0.00	0.00	1.19	0.00	2.03	17.28	0.00	28.86	0.00	1.80	0.41
	**F 35**	10.21	1.96	12.83	0.00	0.00	0.00	0.40	6.78	3.14	11.52	0.43	2.42	4.41	0.00	0.00	0.00	0.00	0.36	0.33	0.00	23.20	0.00	7.16	9.56	0.00	0.00
**Degraded**	**D 3**	7.64	2.21	8.76	0.00	0.00	0.00	0.32	5.15	4.74	5.70	0.31	0.00	0.00	5.41	0.53	0.00	0.53	0.26	2.72	21.16	13.46	0.00	7.37	7.63	0.25	0.00
	**D 4**	3.41	3.05	7.00	0.00	5.29	0.23	6.75	0.00	0.00	11.66	0.00	2.11	3.09	0.00	0.00	0.00	0.86	3.66	4.26	0.00	19.24	0.00	23.98	0.00	0.00	0.32
	**D 6**	4.75	1.74	8.46	0.00	4.23	4.57	4.36	0.00	0.00	5.44	0.95	4.77	3.13	0.16	0.00	0.00	1.00	0.20	11.15	0.12	17.05	0.00	15.42	4.01	2.20	1.89
	**D 7**	3.17	1.34	6.38	1.54	5.45	3.28	0.15	1.35	2.74	5.95	0.83	0.00	3.80	2.92	0.00	0.00	0.00	0.86	7.06	9.32	13.16	0.00	27.29	0.00	1.18	0.60
	**D 8**	6.45	1.61	5.38	0.00	0.63	1.97	0.10	2.05	1.70	8.46	0.05	0.00	1.97	5.75	0.63	0.00	0.60	0.00	0.33	11.89	14.75	0.00	30.02	0.00	0.00	0.00
	**D 9**	4.63	1.55	4.02	0.00	6.16	3.26	0.00	4.83	2.43	5.80	1.25	0.00	0.00	4.20	4.39	0.00	0.00	1.91	3.06	2.39	15.32	0.00	32.33	0.00	0.00	0.35
	**D 10**	4.37	1.39	5.08	0.92	0.00	0.00	0.18	3.51	4.67	6.30	0.34	4.76	0.00	0.00	3.81	0.19	0.31	0.41	5.16	5.34	18.50	0.00	26.55	0.00	0.00	0.89
	**D 11**	4.32	1.19	4.37	0.00	0.00	0.00	0.60	3.84	4.18	7.49	0.52	0.00	3.03	4.87	1.89	0.00	0.31	0.11	0.66	0.66	22.48	0.00	33.95	0.00	0.00	0.49
**Floodplain**	**V 5**	9.11	1.50	0.00	0.00	5.18	6.62	0.48	4.93	0.32	17.68	1.26	1.69	0.76	1.10	0.38	0.83	0.52	0.60	1.64	4.91	12.09	4.04	7.13	7.37	0.00	0.00
	**V 7**	4.76	4.03	0.00	0.00	9.91	1.50	0.53	3.01	3.32	13.12	1.30	0.00	1.01	4.47	0.00	0.00	2.26	0.96	2.34	0.00	26.46	4.98	6.25	6.75	0.08	0.00
	**V 8**	2.22	3.91	4.29	1.07	4.86	5.23	0.22	4.24	6.47	8.46	1.06	0.00	5.44	6.96	0.00	1.48	1.37	2.60	0.21	2.28	13.92	0.00	15.77	0.00	1.56	1.09
	**V 9**	1.50	2.82	0.00	0.00	17.31	3.19	0.18	1.10	0.44	9.17	0.40	0.00	2.79	3.01	0.00	15.41	0.00	0.00	5.59	3.74	0.00	2.97	23.94	0.00	1.01	1.72
	**V 10**	3.12	2.60	9.77	0.17	0.00	0.00	0.25	4.08	12.31	13.50	1.81	0.00	0.00	6.10	7.34	2.72	0.49	0.83	0.20	4.34	1.62	0.52	20.18	0.00	0.17	0.71
	**V 11**	1.71	1.65	7.82	0.00	10.61	2.80	0.59	1.39	7.26	18.15	1.12	0.19	0.71	1.46	5.46	0.32	0.57	0.00	1.46	11.89	0.75	0.00	21.92	0.00	0.00	0.10
	**V 12**	1.23	2.03	17.20	0.00	0.70	0.12	0.33	10.21	1.32	11.49	0.00	0.28	2.23	3.46	0.00	1.38	0.06	0.00	7.69	10.56	8.53	0.00	17.43	0.00	0.00	0.00
	**V 13**	3.02	3.99	0.00	0.00	14.00	0.00	0.88	0.73	5.78	20.93	0.00	0.00	0.00	16.52	2.29	2.20	1.00	0.00	2.42	8.56	0.94	0.00	11.99	0.00	0.00	0.43
	**V 16**	4.05	1.63	0.00	0.00	6.12	0.00	0.00	1.63	1.86	12.40	0.81	0.00	0.00	0.00	4.81	0.00	0.92	0.56	0.80	25.43	0.00	4.01	33.16	0.00	0.00	0.00
**Pasture**	**O 1**	5.50	4.14	4.57	0.20	6.92	0.88	0.00	0.88	0.59	4.49	1.54	1.50	2.21	2.13	0.00	0.00	0.00	0.00	0.82	0.00	25.56	0.00	35.50	0.00	0.00	0.00
	**O 2**	5.19	4.28	4.19	0.00	6.25	0.92	0.00	0.68	0.48	4.11	1.13	1.30	2.39	2.50	0.00	0.00	0.00	0.00	0.52	0.00	26.23	0.00	37.75	0.00	0.00	0.00
	**O 3**	8.11	1.81	0.00	0.00	2.04	2.48	0.20	0.00	4.87	7.69	0.49	2.13	2.68	3.42	0.00	0.00	0.00	0.00	1.77	0.00	26.34	0.00	35.31	0.00	0.00	0.00
	**O 5**	7.88	1.76	0.00	0.00	2.06	2.92	0.00	0.00	3.54	6.20	0.58	0.00	3.08	3.52	0.00	0.00	0.50	0.00	3.31	0.00	26.73	0.00	36.31	0.00	0.00	0.00
	**O 6**	5.89	2.46	1.99	0.00	9.85	2.06	0.00	0.53	0.00	0.45	3.86	2.22	2.09	5.84	0.00	0.00	0.81	2.31	1.42	0.24	23.13	0.00	31.52	0.00	0.42	0.69
	**O 7**	5.47	1.98	4.40	0.00	5.84	1.85	0.00	0.23	0.00	5.12	1.05	2.86	1.38	4.13	0.00	0.00	0.79	1.80	0.56	0.00	26.66	0.00	33.27	0.00	0.00	0.15
	**O 9**	4.89	2.44	2.84	1.14	17.82	3.51	0.00	2.30	0.91	0.00	3.46	0.00	6.70	6.16	0.00	1.34	0.27	0.21	5.08	0.29	11.00	2.16	24.22	0.00	1.12	0.00
	**O 15**	5.32	1.27	2.11	0.00	7.27	0.00	0.00	0.00	0.00	4.20	1.18	4.71	16.76	2.29	1.29	0.00	0.40	0.00	2.19	0.20	25.50	0.00	21.71	0.00	0.18	0.00
	**O 16**	4.79	2.18	5.99	0.17	1.17	0.96	0.08	2.53	0.00	8.06	0.00	7.32	1.98	5.93	0.00	0.00	0.41	0.00	5.71	0.00	20.37	0.00	30.20	0.00	0.14	0.39
	**O 19**	4.84	0.57	1.23	0.00	12.43	0.00	0.40	1.15	0.00	9.93	0.00	1.53	6.13	1.60	0.29	0.00	0.00	0.00	2.30	1.25	19.40	0.00	30.30	0.00	0.32	0.23

Gradual scales in green or red represent values above or below the average of peak area, respectively.

## Supplementary Materials

The following are available online at https://www.mdpi.com/article/10.3390/toxins13110814/s1, Figure S1: Chromatographic profiles of *B. atrox* venoms of different habitats from west of Pará State, Brazilian Amazon; Figure S2: Characteristics and localization of areas of snake collection.
