# Supplementary material for: Individual Variability in Bothrops atrox Snakes Collected from Different Habitats in the Brazilian Amazon: New Findings on Venom Composition and Functionality
Source: Toxins (Basel). 2021 Nov 18;13(11):814. doi: 10.3390/toxins13110814 (PMC8618853; doi:10.3390/toxins13110814)
Supplement: Supplementary file 1 [file toxins-13-00814-s001.zip › toxins-1399633-supplementary.pdf]

Supplementary Materials: Individual Variability in *Bothrops atrox* Snakes Collected from Different Habitats in the Brazilian Amazon: New Findings on Venom Composition and Functionality

Leijane F. Sousa, Matthew L. Holding, Tiago H. M. Del-Rei, Marisa M. T. Rocha, Rosa H. V. Mourão, Hipócrates M. Chalkidis, Benedito Prezoto, H. Lisle Gibbs and Ana M. Moura-da-Silva

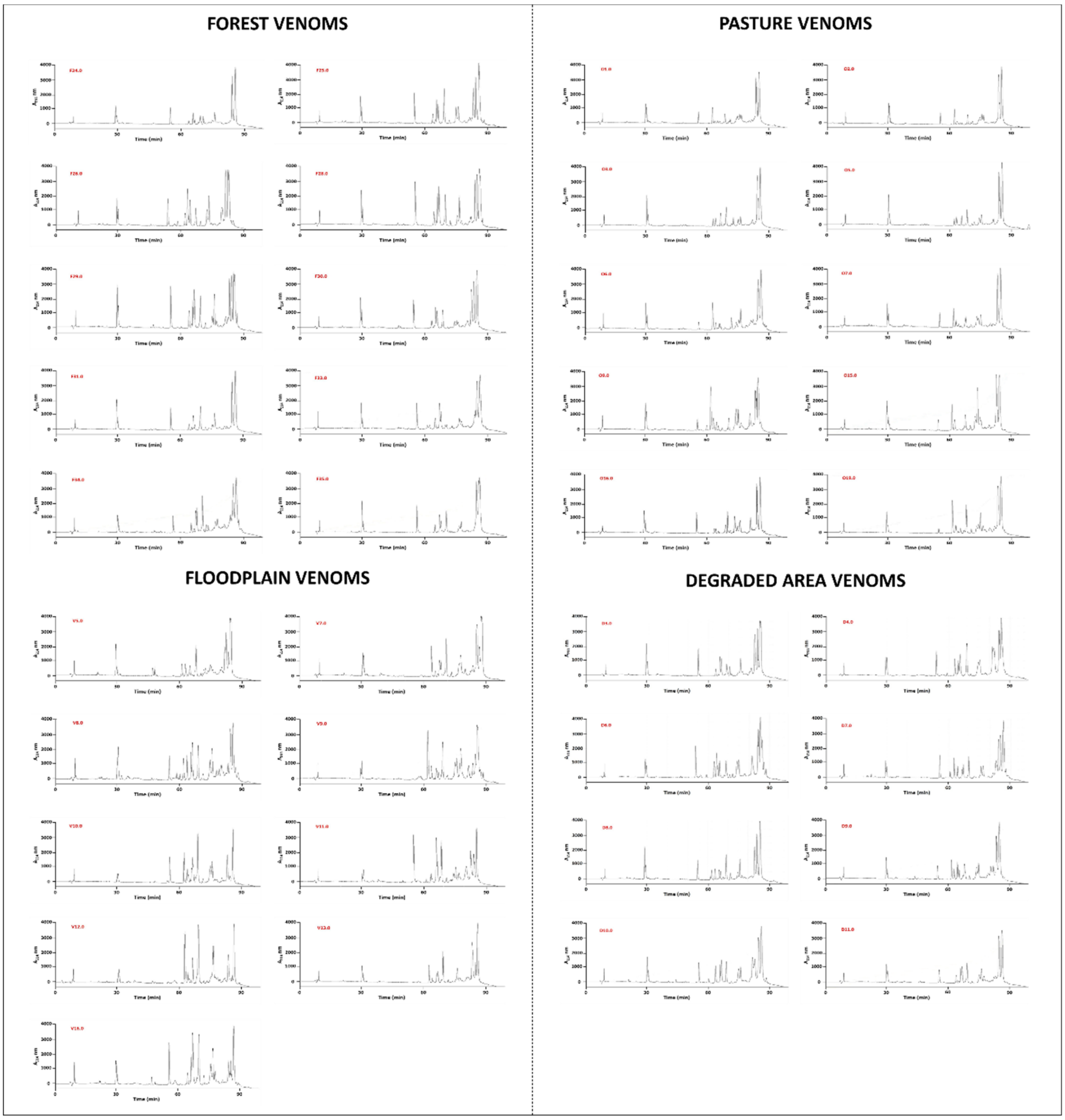

Figure S1. Chromatographic profiles of B. atrox venoms of different habitats from west of Pará State, Brazilian Amazon. Individual venom samples (5 mg) of B. atrox snakes collected at the forest, pasture, degraded area, or floodplain were applied to a Vydac C-18 column. Mobile phases used were 0.1% TFA in water (A) or 0.1% TFA in acetonitrile (B). Proteins were gradient-eluted at 2 mL/min (5% B for 5 min, 5–15% B over 10 min, 15–45% B over 60 min, 45–70% B over 10 min, 70–100% over 5 min, and 100% B over 10 min). Separation was monitored at 214 nm.

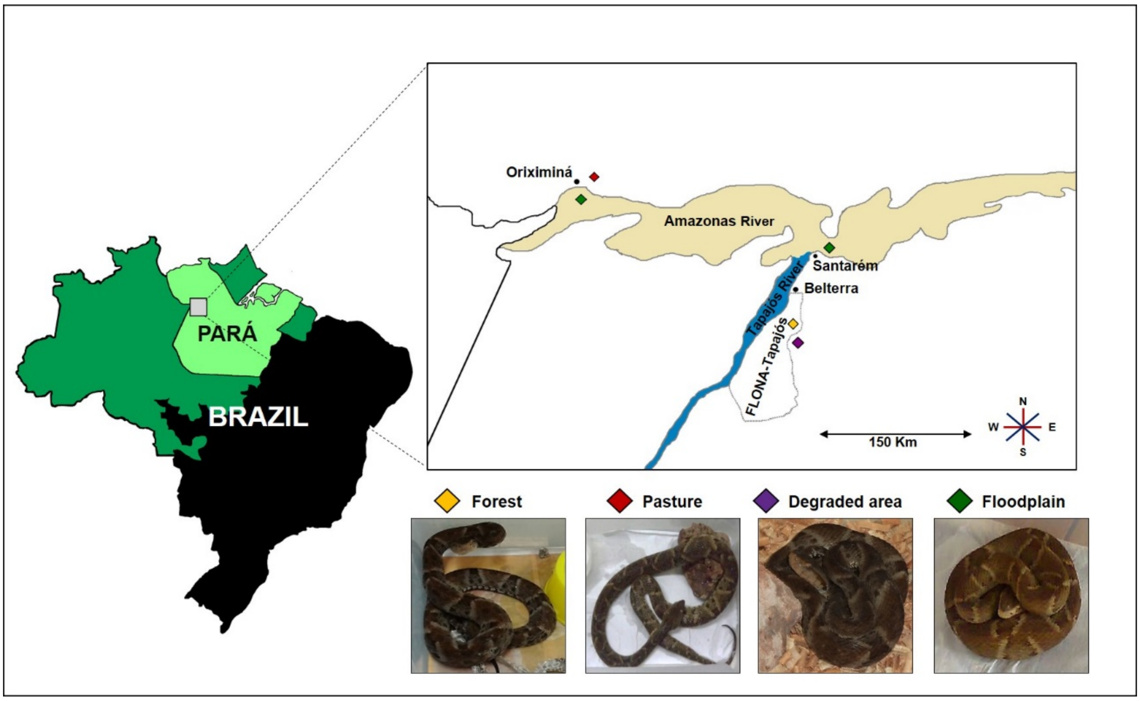

Figure S2. Areas of snake collection at Pará State, Brazilian Amazon. Brazilian map in black highlighting the Brazilian Amazon (dark green), Pará State (light green) and the collection sites in the west this State (light gray). The amplification of the square denotes the location of the areas of snake collection: pasture (red) at the north shore of the Amazon River, municipality of Oriximiná; forest (yellow) and degraded area (purple) at Belterra municipality, eastern shore of the Tapajós River; floodplain regions in Santarém and Oriximiná municipalities. Photos show specimens of *B. atrox* captured in each habitat. Photos by: Chalkidis,HM.
